# Marchiafava-Bignami disease with rare etiology: A case report

**DOI:** 10.3892/etm.2015.2263

**Published:** 2015-02-05

**Authors:** YONGJIAN CUI, LEI ZHENG, XIAOLI WANG, WEIWEN ZHANG, DONGCAI YUAN, YAN WEI

**Affiliations:** 1 The Second Department of Neurology, Harrison International Peace Hospital, Hengshui, Hebei 053000, P.R. China; 2 Central Laboratory, Harrison International Peace Hospital, Hengshui, Hebei 053000, P.R. China

**Keywords:** Marchiafava-Bignami disease, chronic alcoholism, non drinkers, cardia carcinoma, malnutrition

## Abstract

A male, 62-year-old patient was admitted to hospital due to dizziness and gait disturbance for 10 days. The patient had fallen a few times due to the gait instability, which was associated with stiffness and memory loss. The patient had undergone cardiac carcinoma surgery three years previously and had no drinking history. Physical examination revealed that the patient was lucid when conscious but exhibited slurred speech, apathy and cognitive impairment. The finger-to-nose and rapid alternating movement tests showed the patient to be slightly clumsy. Magnetic resonance imaging revealed symmetric abnormal signals in the splenium of the corpus callosum, and the diagnosis was Marchiafava-Bignami disease (MBD). The patient recovered following the administration of vitamin B and other treatments. The patient had long-term appetite loss. A brain myelin metabolism disorder caused by long-term malnutrition and leading to demyelinating changes in the brain may have been the cause of the MBD of this patient. Clinicians should increase awareness of this disease and should not ignore the diagnosis of it, even when the patient lacks a drinking history. Early diagnosis and treatment can improve the prognosis of the patient.

## Introduction

Marchiafava-Bignami disease (MBD) is a rare neurological disease often associated with chronic, heavy alcohol consumption and malnutrition, and is characterized by callosal lesions consisting of necrosis and demyelination (1–4). Over the past few years, magnetic resonance imaging (MRI) findings of the callosal and cortical lesions, which are critical for the diagnosis of MBD, have been investigated (5). To date, the etiology of MBD is incompletely understood. In excess of 90% of patients with MBD exhibit a poor prognosis (6); however, with adequate therapy, these patients can recover completely, with the disappearance of the callosal lesions on serial MRI (7–8).

It is well known that MBD is widely observed and can be caused by any alcoholic beverage. It appears that, with alcoholism, the prognosis is worse; however, MBD can occur in patients who have no history of alcohol abuse (9–11). The main hypothesis for the pathogenesis of MBD is that the condition is associated with malnutrition or vitamin B deficiency (9), although there are reports describing cases of MBD caused by existing cyanide, CO poisoning and sepsis, as well as sickle cell disease and *Plasmodium* infection (3). The treatment of MBD in those patients remains controversial; however, the administration of nutritional factors, such as high-dose group B vitamins and folic acid, is believed to be beneficial, and numerous patients have shown improvements following the administration of vitamin B therapy (9,12).

The present study describes a case of MBD in a non-drinker, who had previously undergone cardiac carcinoma surgery. The study was conducted in accordance with the Declaration of Helsinki and with approval from the Ethics Committee of the Harrison International Peace Hospital (Hengshui, China). Written informed consent was obtained from participant.

## Case report

### Patient history

The patient, a 62-year-old male, came from Hengshui Wuyi county and was referred to our department due to dizziness and gait instability that had persisted for >10 days. The patient staggered from side to side, fell several times due to the gait instability, did not dare to stand and exhibited continuously worsening symptoms. The patient also had a dull expression and hypomnesia. Three years previously, the patient had undergone cardiac carcinoma surgery and was prescribed long-term oral ranitidine and furazolidone. The family members of the patient complained that his food intake had decreased significantly and that he had recently suffered from delusions, following which his appetite had reduced further. The patient had no history of poison contact or drinking or drug abuse.

### Physical, biochemical and imaging examinations

**Figure 1:**
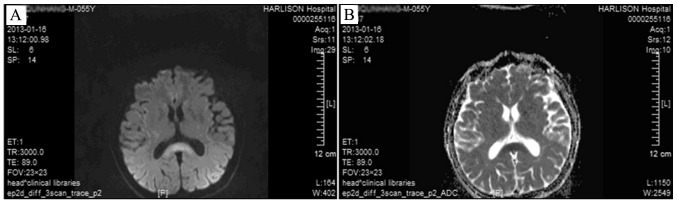
Magnetic resonance imaging showed symmetric abnormal signals in the splenium of the corpus callosum. (A) Diffusion-weighted imaging sequences showed a high signal in the splenium of the corpus callosum. (B) Apparent diffusion coefficient sequences showed a low signal in the splenium of the corpus callosum.

Physical examination of the patient revealed that he was lucid when conscious, but exhibited slurred speech, apathy, cognitive impairment and poor calculation and memory. The bilateral pupils of the patient were round and equal, his light reflex and eyeball motion were normal, and the patient did not exhibit nystagmus. His bilateral frontalis and nasolabial groove were approximately symmetrical, and his tongue was in the center. The muscle strength of the patient’s extremities was grade 5, with normal muscle tone. The patient had no sensory disturbance, and his physiological reflexes were present without pathological reflex. The finger-to-nose and fast alternating movement tests showed the patient to be slightly clumsy, and MRI showed symmetric abnormal signals in the splenium of the corpus callosum (Fig. 1). Blood lipid tests revealed a total cholesterol level of 6.29 mmol/l and a low-density lipoprotein level of 3.96 mmol/l. Routine blood, urine, liver and kidney function and blood coagulation tests showed no obvious abnormalities, and no abnormalities were found with thoracic and abdominal computed tomography. The diagnosis was MBD. The patient was told to consume a diet rich in vitamins to improve the brain blood and oxygen supply, and was prescribed vitamins B1 and B6, methylcobalamin, and folic acid treatments. Two weeks after admission, the slow responses and delusions of the patient had improved markedly. Following discharge, the patient was followed-up for two months. He could walk freely and live on his own.

## Discussion

To date, the etiology of MBD is incompletely understood, as it is one of the rare complications of chronic alcoholism (1–2). It is believed that MBD may be closely associated with chronic alcoholism and/or malnutrition caused by long-term alcohol abuse; however, MBD can also occur in patients who have no history of alcohol abuse (9–11). The main hypothesis for MBD pathogenesis is that the condition may be associated with malnutrition or vitamin B deficiency (9), although it is believed that MBD may have other etiologies. There are reports on MBD caused by existing cyanide, CO poisoning and sepsis, as well as sickle cell disease and *Plasmodium* infection (3).

The present study describes a patient who had undergone cardiac carcinoma surgery three years previously, and who had received chemotherapy three times; the last chemotherapy session was one-and-a-half years prior to admission. The etiology of the patient’s condition may have been similar to that described in a previous study, in which a metabolic disorder of the myelin in the brain of a patient with no long-term alcohol abuse, which was caused by long-term malnutrition involving protein, folic acid and particularly thiamine deficiency, led to demyelinating changes and MBD (9). The corpus callosum, which is the largest commissural fiber system within the hemisphere, has a relatively high myelin content, and may often cause the degeneration and necrosis of nerve cells in such patients. The treatment of MBD in these patients remains controversial, although it is believed that nutritional factors, such as high-dose group B vitamins and folic acid, may be beneficial.

Over the past few years, MRI findings of the callosal and cortical lesions, which are critical for the diagnosis of MBD, have been investigated (5). An improvement in the neurological disorder occurring concurrently with a change in the MRI findings following therapy should therefore enhance the understanding of the disease, which may be beneficial for the definitive diagnosis and monitoring of MBD in patients with no long-term alcohol abuse. Specific MRI changes have been approved as the main basis for the diagnosis of the condition, including a slightly low or hypointense signal on the T1WI, and a hyperintense signal on the T2WI, fluid-attenuated inversion recovery and diffusion-weighted imaging sequences. The MR images of the patient in the present case were consistent with those expected for MBD (Fig. 1A and B).

For the patient in the present report, a number of diseases and conditions had to be excluded prior to a diagnosis of MBD being reached. The first condition was paraneoplastic syndrome (PNS), which is characterized by symptoms including cerebellar degeneration and limbic encephalitis. Patients with PNS also exhibit cerebrospinal fluid (CSF) pleocytosis and elevated protein and immunoglobulin G levels. The routine CSF and biochemical tests of the present patient were normal, and the brain MRI results were not consistent with PNS. Furthermore, the condition of the patient improved following vitamin treatment; none of these observations supported a diagnosis of PNS. Secondly, multiple sclerosis (MS) had to be excluded. MS lesions are relatively small, and the condition predominantly manifests as multiple lesions of the periventricular white matter. The lesions rarely develop initially or appear only in the corpus callosum. In the majority of cases of MS, the condition exhibits remission and relapse. In the present case, the condition of the patient, the CSF tests and the MRI findings were not in accordance with MS. A third possible condition was Wernicke’s encephalopathy (WE). In patients with WE, the bilateral thalamus and brainstem often show symmetrical lesions, and the typical MRI findings are abnormal signals in the mammillary body, aqueduct and around the third ventricle, which can be combined with MBD. The MRI of this patient revealed a solitary lesion in the corpus callosum, which was not consistent with WE. A fourth condition to be excluded was corpus callosum infarction, which is rarely seen in the clinic. The anterior and posterior cerebral arteries supply blood to the anterior and posterior corpus callosum, respectively, and most lesions are unilateral. A fifth possible condition was progressive multifocal leukoencephalopathy (PML), which is a type of progressive demyelinating disease caused by papovavirus infection. In addition to the corpus callosum, this disease involves the subcortical white matter, and the lesions show a non-symmetrical distribution. Furthermore, as time progresses, the lesions may exhibit an integration trend. PML is dangerous, progressive and has a poor prognosis, but the observations in the present case did not support a PML diagnosis. Finally, a poison-induced disease had to be excluded. Since the mechanisms underlying brain damage in poisoning diseases, such as CO, organophosphorus, lead or mercury poisoning, are different, the lesions in the cerebral white matter are different, and the MRI features, which involve the basal ganglia, brainstem, cerebellum and cortex, are not specific. Furthermore, the patient may have a clear history of poisoning.

MBD may be a reversible brain disease. Clinicians should enhance the awareness of the disease and emphasize that the disease cannot be ignored in patients without a drinking history, particularly in malnourished patients. An improvement in the neurological disorder occurring concurrently with a change in the corpus callosum degeneration following therapy should enhance the understanding of the disease, which may be beneficial for the definitive diagnosis and early treatment of MBD.
